# Crystal structure of memantine–carb­oxy­borane

**DOI:** 10.1107/S2056989019004092

**Published:** 2019-04-02

**Authors:** Theppawut I. Ayudhya, Arnold L. Rheingold, Nin N. Dingra

**Affiliations:** a Department of Chemistry, University of Alaska Anchorage, Anchorage, AK 99508, USA; b Department of Chemistry, University of California–San Diego, La Jolla, CA 92093, USA

**Keywords:** crystal structure, memantine-carb­oxy­borane, CORCB, memantine, adamantane

## Abstract

The synthesis and crystal structure of the title compound, C_13_H_24_BNO_2_ [systematic name: 3,5-di­methyl­adamantanyl­amine–borane­carb­oxy­lic acid or *N*-(carb­oxy­boranyl­idene)-3,5-di­methyl­adamantan-1-amine], derived from the anti-Alzheimer’s disease drug memantine is reported. The C—N—B—CO_2_ unit is almost planar (r.m.s. deviation = 0.095 Å). The extended structure shows typical carb­oxy­lic acid inversion dimers linked by pairwise O—H⋯O hydrogen bonds [O⋯O = 2.662 (3) Å]. The amino group forms a weak N—H⋯O hydrogen bond [N⋯O = 3.011 (3) Å], linking the dimers into [001] chains in the crystal. Highly disordered solvent mol­ecules were treated using the SQUEEZE routine of *PLATON* [Spek (2015 ▸). *Acta Cryst*. C**71**, 9–18], which treats the electron density as a diffuse contribution without assignment of specific atom locations. A scattering contribution of 255 electrons was removed. The crystal studied was refined as a two-component twin.

## Abstract 2

The crystal structure of a modified anti-Alzheimer’s drug features O—H⋯O and N—H⋯O hydrogen bonds.

## Chemical context   

Memantine is a drug used for the treatment of mild and moderate-to-severe Alzheimer’s disease as an inhibitor for *N*-methyl-d-aspartate (NMDA) receptors. As a result of its property as a low-affinity, open-channel blocker, memantine does not substanti­ally inter­fere with normal synaptic activity, thereby reducing side effects. This has led to clinical trials for other neurological disorders (Bullock, 2006 ▸; Lipton, 2005 ▸; Olivares *et al.*, 2012 ▸; Parsons *et al.*, 2007 ▸). While memantine in its hydro­chloride form is useful in various treatment methods, some modifications were done on this drug to optimize the desired concentration in the system. As a means to preventing drug degradation, memantine has been further processed in a mixture with other compounds (McInnes *et al.*, 2010 ▸; Plosker, 2015 ▸). The one-week extended release formula by Lyndra Therapeutics is currently under clinical trial phase I (clinicaltrials.gov, NCT03711825). Though efforts to maintain the long-term stability of memantine are underway, chemical modification of the memantine structure itself is rarely reported. Our attempt was to mask the compound with an additional moiety that can be removed under certain conditions, therefore releasing the drug. With this goal, memantine–carb­oxy­borane was synthesized since the carb­oxy­borate group is known to decompose into carbon monoxide and boric acid, leaving the drug mol­ecule itself (Ayudhya *et al.*, 2017 ▸, 2018 ▸). The single crystal structure of the said compound, (I), was solved and its features are described in this report.

## Structural commentary   

**Figure 1:**
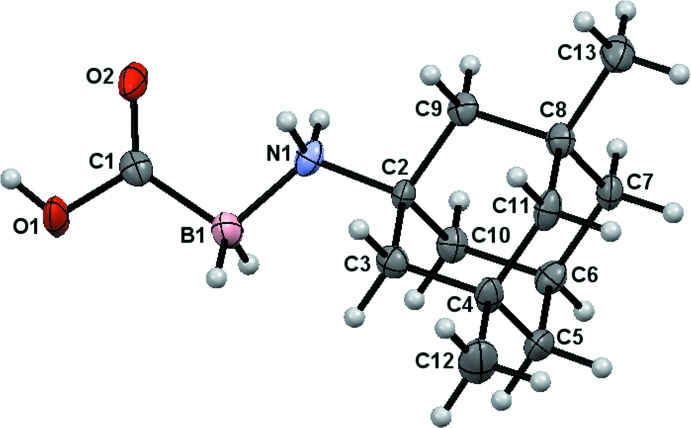
The mol­ecular structure of (I) with displacement ellipsoids drawn at the 50% probability level.

The mol­ecular structure of (I) is shown in Fig. 1 ▸. The C2–N1–B1–C1/O1/O2 fragment is almost planar (r.m.s. deviation = 0.095 Å) and the C atoms bonded to the B and N atoms take on an anti orientation [C1—B1—N1—C2 = 173.5 (3)°]. The stereogenic centres in the adamantane unit were assigned as C4 *S* and C8 *R* in the arbitrarily chosen asymmetric unit but crystal symmetry generates a racemic mixture. The bond lengths [C1—O1 = 1.340 (4), C1—O2 = 1.227 (4) Å] of the carb­oxy­lic acid group are in agreement with the data for related carb­oxy­lic acids and known amine–carb­oxy­boranes (Gavezzotti, 2008 ▸; Spielvogel *et al.*, 1980 ▸; Vyakaranam *et al.*, 2002 ▸; Ayudhya *et al.*, 2017 ▸). The C—C—C bond angles of the adamantine cage fall within the expected ranges and the N1—C2 bond length at 1.504 (4) Å is comparable with previously reported values in amino­adamantane structures (Donohue & Goodman, 1967 ▸; Chacko & Zand, 1973 ▸).
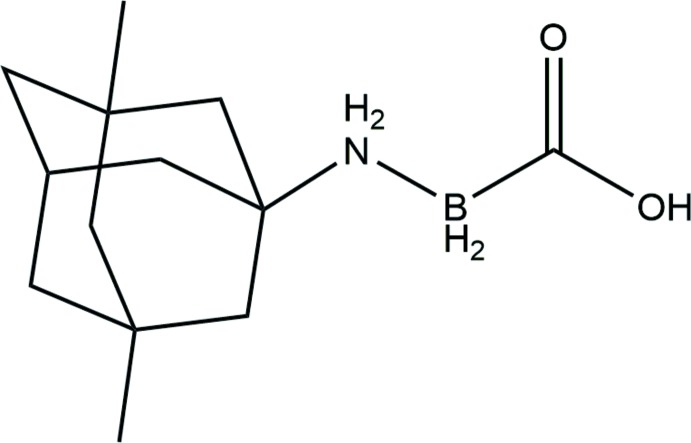


## Supra­molecular features   

**Figure 2:**
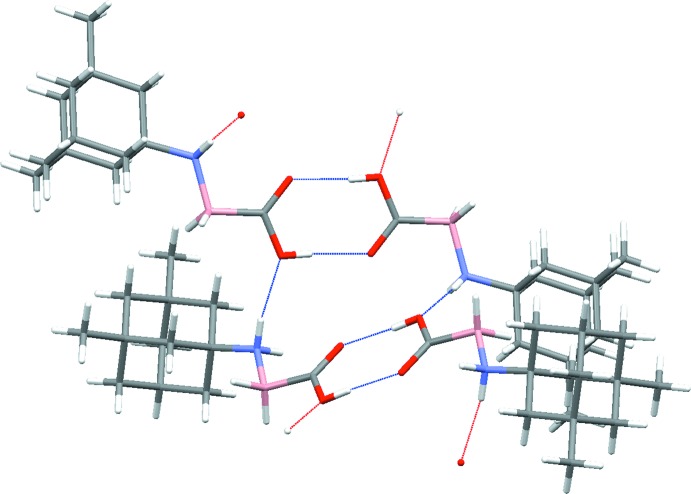
Detail of the hydrogen bonds in (I) showing the carb­oxy­lic acid inversion dimers and N—H⋯O links between dimers.

A dimer is observed between the two memantine–carb­oxy­borane mol­ecules formed through conventional hydrogen bonding between the carb­oxy­lic acid moieties (Fig. 2 ▸). The hydrogen-bond length listed in Table 1 ▸ [O1⋯O2 = 2.662 (3) Å] is consistent with the hydrogen-bond geometries found in carb­oxy­borane dimers such as ammonia–carb­oxy­borane [2.668 (2) Å; Spielvogel *et al.*, 1980 ▸], mophorline–carb­oxy­borane [2.712 (4) Å; Vyakaranam *et al.*, 2002 ▸] and tri­methyl­amine–carb­oxy­borane [2.714 Å; Spielvogel *et al.*, 1976 ▸]. In (I), these dimers form an extended structure through N1—H1*B*⋯O1 links (O1 is the protonated oxygen atom of the carb­oxy­lic acid), to form [001] chains. This motif has also been reported previously in ammonia–carb­oxy­borane, tri­methyl­amine–carb­oxy­borane, di­methyl­amine–carb­oxy­borane and methyl­amine–carb­oxy­borane (Spielvogel *et al.*, 1980 ▸). The adjacent dimers shown in Fig. 2 ▸ indicates that the planes of the carb­oxy­lic acids are not parallel, but twisted by 76.5° from each other.

**Figure 3:**
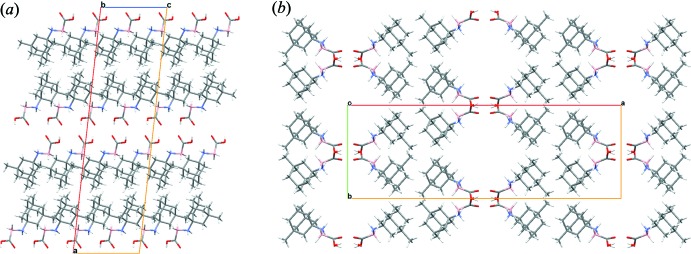
Packing diagrams of (I): (*a*) A view from the *b* axis to show aligned hydrogen-bonding dimers. (*b*) A view down the *c* axis to show the twisted planes.

Assessment of available crystal structures deposited with the Cambridge Structural Database (Version 5.39; Groom *et al.*, 2016 ▸) indicates that not all amine–carb­oxy­boranes form dimers during crystallization. While some amine–carb­oxy­boranes described above are dimers, others such as piperidine–carb­oxy­borane and hexa­methyl­ene­tetra­mine–carb­oxy­borane do not form dimers, suggesting that the amine-group inter­action may influence the overall packing (Rana *et al.*, 2002 ▸; Ayudhya *et al.*, 2017 ▸). The extended structure of (I) is shown in projection down the *b-* and *c*-axis directions in Fig. 3 ▸ *a* and 3*b*, respectively. No other contacts beyond the hydrogen bonds already mentioned are observed in this packing. Although the dimers appear to be parallel in Fig. 3 ▸ *a*, the twisted planes of hydrogen bonds are better represented in Fig. 3 ▸ *b*.

## Database survey   

The memantine structure in its free (unprotonated) base form is not found in the literature, although the hydro­chloride salt with water mol­ecules of crystallization has been solved (Lou *et al.*, 2009 ▸). Another memantine crystal structure reported was in a clathrate form with cucurbit[7]uril where memantine is completely bound within the cavity (McInnes *et al.*, 2010 ▸). However, numerous crystal structures of the adamantane cage and its derivatives in various forms have been reported over many years (Nordman & Schmitkons, 1965 ▸; Chacko & Zand, 1973 ▸; SiMa, 2009 ▸; Glaser *et al.*, 2011 ▸).

## Synthesis and crystallization   

**Figure 4:**
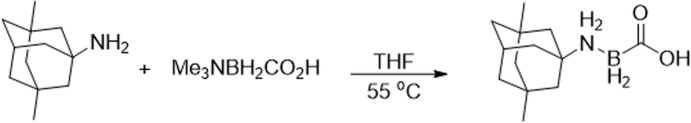
Reaction scheme for the synthesis of (I) through an amine-exchange process.

Memantine, a derivative of adamantine, was first synthesized by Eli Lilly and Company. In an attempt to modify memantine into memantine–carb­oxy­borane, a reaction scheme as shown in Fig. 4 ▸ was carried out. Addition of the carb­oxy­borane moiety to memantine was done in a one-step reaction using an amine-exchange process as previously described (Spielvogel *et al.*, 1980 ▸). Tri­methyl­amine carb­oxy­borane (117 mg, 1.0 mmol) and memantine (780 mg, 4.4 mmol) were dissolved in tetra­hydro­furan (8.0 ml), and maintained at 328 K for 24 h under a nitro­gen atmosphere. The solution was concentrated by vacuum distillation and the resulting solid was dissolved in di­chloro­methane. The product was precipitated from the solvent by using 15 ml of hexane and the white solid crude product (208 mg) was filtered. This residue was purified by multiple recrystallization in di­chloro­methane/hexane to yield a white solid (15 mg, 6.3%). Crystals suitable for X-ray analysis were prepared by dissolving in toluene and slow cooling of the solution.

## Refinement   

Crystal data collection and structure refinement details are summarized in Table 2 ▸. H atoms were placed in calculated positions (O—H = 0.84, N—H = 0.91 and C—H = 0.98–0.99 Å) and refined as riding with *U*_iso_(eq) = 1.5*U*_eq_(C-methyl, O) and 1.2*U*_eq_(C, N) for all others. The idealized methyl groups at C12 and C13 and the idealized tetra­hedral OH group at O1 were refined as rotating groups. The disordered solvent mol­ecules were treated with the SQUEEZE routine in PLATON (Spek, 2015 ▸). The crystal studied was refined as a two-component twin.

## Supplementary Material

Crystal structure: contains datablock(s) I. DOI: 10.1107/S2056989019004092/hb7806sup1.cif

Structure factors: contains datablock(s) I. DOI: 10.1107/S2056989019004092/hb7806Isup2.hkl

Supporting information file. DOI: 10.1107/S2056989019004092/hb7806Isup4.cdx

Supporting information file. DOI: 10.1107/S2056989019004092/hb7806Isup5.cdx

Graphical Abstract. DOI: 10.1107/S2056989019004092/hb7806sup3.tif

Supporting information file. DOI: 10.1107/S2056989019004092/hb7806Isup6.cml

CCDC reference: 1905840

Additional supporting information:  crystallographic information; 3D view; checkCIF report

## Figures and Tables

**Table 1:** Hydrogen-bond geometry (Å, °)

*D*—H⋯*A*	*D*—H	H⋯*A*	*D*⋯*A*	*D*—H⋯*A*
O1—H1⋯O2^i^	0.84	1.82	2.662 (3)	176
N1—H1*B*⋯O1^ii^	0.91	2.11	3.011 (3)	171

Symmetry codes: (i) 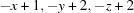; (ii) 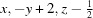.

**Table 2:** Experimental details

Crystal data	
Chemical formula	C_13_H_24_BNO_2_
*M*_r_	237.14
Crystal system, space group	Monoclinic, *C*2/*c*
Temperature (K)	100
*a*, *b*, *c* (Å)	34.229 (4), 11.1051 (12), 9.2922 (10)
β (°)	96.526 (5)
*V* (Å^3^)	3509.3 (7)
*Z*	8
Radiation type	Mo *K*α
μ (mm^−1^)	0.06
Crystal size (mm)	0.32 × 0.30 × 0.10

Data collection	
Diffractometer	Bruker APEXII Ultra
Absorption correction	Multi-scan (*TWINABS*; Bruker, 2012 ▸)
*T*_min_, *T*_max_	0.300, 0.333
No. of measured, independent and observed [*I* > 2σ(*I*)] reflections	10740, 10740, 8531
(sin θ/λ)_max_ (Å^−1^)	0.611

Refinement	
*R*[*F* ^2^ > 2σ(*F* ^2^)], *wR*(*F* ^2^), *S*	0.062, 0.154, 1.04
No. of reflections	10740
No. of parameters	166
H-atom treatment	H atoms treated by a mixture of independent and constrained refinement
Δρ_max_, Δρ_min_ (e Å^−3^)	0.94, −0.24

Computer programs: *APEX3* and *SAINT* (Bruker, 2017 ▸), *SHELXT* (Sheldrick, 2015*a* ▸), *SHELXL2014* (Sheldrick, 2015*b* ▸) and *OLEX2* (Dolomanov *et al.*, 2009 ▸).

## supplementary crystallographic information

### Crystal data

**Table:**

C_13_H_24_BNO_2_	*F*(000) = 1040
*M**_r_* = 237.14	*D*_x_ = 0.898 Mg m^−^^3^
Monoclinic, *C*2/*c*	Mo *K*α radiation, λ = 0.71073 Å
*a* = 34.229 (4) Å	Cell parameters from 1799 reflections
*b* = 11.1051 (12) Å	θ = 2.9–25.6°
*c* = 9.2922 (10) Å	µ = 0.06 mm^−^^1^
β = 96.526 (5)°	*T* = 100 K
*V* = 3509.3 (7) Å^3^	Plate, colourless
*Z* = 8	0.32 × 0.30 × 0.10 mm

### Data collection

**Table:**

Bruker APEXII Ultra diffractometer	10740 measured reflections
Radiation source: Micro Focus Rotating Anode, Bruker TXS	10740 independent reflections
Double Bounce Multilayer Mirrors monochromator	8531 reflections with *I* > 2σ(*I*)
Detector resolution: 8.258 pixels mm^-1^	θ_max_ = 25.7°, θ_min_ = 1.2°
φ and ω scans	*h* = −41→41
Absorption correction: multi-scan (*TWINABS*; Bruker, 2012)	*k* = −13→13
*T*_min_ = 0.300, *T*_max_ = 0.333	*l* = −11→11

### Refinement

**Table:**

Refinement on *F*^2^	Primary atom site location: dual
Least-squares matrix: full	Hydrogen site location: mixed
*R*[*F*^2^ > 2σ(*F*^2^)] = 0.062	H atoms treated by a mixture of independent and constrained refinement
*wR*(*F*^2^) = 0.154	*w* = 1/[σ^2^(*F*_o_^2^) + (0.0598*P*)^2^ + 6.9836*P*] where *P* = (*F*_o_^2^ + 2*F*_c_^2^)/3
*S* = 1.04	(Δ/σ)_max_ < 0.001
10740 reflections	Δρ_max_ = 0.94 e Å^−^^3^
166 parameters	Δρ_min_ = −0.24 e Å^−^^3^
0 restraints	

### Special details

**Table:**

Geometry. All esds (except the esd in the dihedral angle between two l.s. planes) are estimated using the full covariance matrix. The cell esds are taken into account individually in the estimation of esds in distances, angles and torsion angles; correlations between esds in cell parameters are only used when they are defined by crystal symmetry. An approximate (isotropic) treatment of cell esds is used for estimating esds involving l.s. planes.
Refinement. Refined as a two-component twin.

### Fractional atomic coordinates and isotropic or equivalent isotropic displacement parameters (Å^2^)

**Table:**

	*x*	*y*	*z*	*U*_iso_*/*U*_eq_	
O1	0.54884 (6)	0.9933 (2)	1.1021 (3)	0.0296 (6)	
H1	0.5259	1.0205	1.0994	0.044*	
O2	0.52308 (7)	0.9168 (2)	0.8929 (2)	0.0373 (7)	
N1	0.59334 (7)	0.8289 (2)	0.8033 (3)	0.0214 (6)	
H1A	0.5763	0.7656	0.7974	0.026*	
H1B	0.5825	0.8865	0.7416	0.026*	
C9	0.61930 (9)	0.7507 (3)	0.5864 (3)	0.0203 (7)	
H9A	0.5993	0.6859	0.5806	0.024*	
H9B	0.6077	0.8209	0.5313	0.024*	
C2	0.63037 (8)	0.7867 (3)	0.7461 (3)	0.0172 (7)	
C10	0.64666 (9)	0.6768 (3)	0.8315 (4)	0.0224 (7)	
H10A	0.6267	0.6120	0.8257	0.027*	
H10B	0.6536	0.6983	0.9346	0.027*	
C8	0.65553 (9)	0.7071 (3)	0.5192 (3)	0.0213 (7)	
C11	0.68651 (9)	0.8083 (3)	0.5324 (4)	0.0241 (8)	
H11A	0.7103	0.7806	0.4906	0.029*	
H11B	0.6759	0.8793	0.4764	0.029*	
C6	0.68384 (9)	0.6335 (3)	0.7642 (4)	0.0229 (8)	
H6	0.6951	0.5618	0.8193	0.027*	
C1	0.55232 (10)	0.9311 (3)	0.9806 (4)	0.0219 (8)	
C4	0.69762 (9)	0.8445 (3)	0.6898 (4)	0.0243 (8)	
C3	0.66090 (9)	0.8865 (3)	0.7553 (4)	0.0222 (7)	
H3A	0.6681	0.9095	0.8578	0.027*	
H3B	0.6498	0.9583	0.7023	0.027*	
C7	0.67253 (9)	0.5977 (3)	0.6057 (4)	0.0225 (7)	
H7A	0.6528	0.5323	0.6002	0.027*	
H7B	0.6960	0.5676	0.5639	0.027*	
C5	0.71424 (9)	0.7332 (3)	0.7751 (4)	0.0277 (8)	
H5A	0.7383	0.7050	0.7354	0.033*	
H5B	0.7214	0.7551	0.8780	0.033*	
C13	0.64387 (10)	0.6749 (3)	0.3590 (4)	0.0298 (9)	
H13A	0.6244	0.6099	0.3522	0.045*	
H13B	0.6672	0.6484	0.3157	0.045*	
H13C	0.6326	0.7458	0.3071	0.045*	
C12	0.72819 (10)	0.9460 (3)	0.6973 (5)	0.0397 (10)	
H12A	0.7515	0.9185	0.6543	0.060*	
H12B	0.7358	0.9683	0.7988	0.060*	
H12C	0.7169	1.0162	0.6438	0.060*	
B1	0.59514 (12)	0.8823 (4)	0.9644 (4)	0.0295 (10)	
H1C	0.6174 (10)	0.964 (3)	0.966 (4)	0.042 (10)*	
H1D	0.6026 (11)	0.810 (3)	1.038 (4)	0.050 (11)*	

### Atomic displacement parameters (Å^2^)

**Table:**

	*U*^11^	*U*^22^	*U*^33^	*U*^12^	*U*^13^	*U*^23^
O1	0.0235 (12)	0.0335 (13)	0.0342 (14)	−0.0008 (11)	0.0140 (12)	−0.0136 (11)
O2	0.0334 (14)	0.0620 (17)	0.0167 (12)	0.0240 (13)	0.0038 (12)	−0.0048 (13)
N1	0.0202 (14)	0.0169 (13)	0.0280 (15)	0.0034 (11)	0.0062 (12)	0.0043 (12)
C9	0.0237 (18)	0.0150 (16)	0.0222 (17)	0.0018 (13)	0.0026 (15)	0.0029 (15)
C2	0.0149 (16)	0.0182 (16)	0.0190 (16)	0.0039 (13)	0.0043 (14)	−0.0018 (14)
C10	0.0236 (18)	0.0213 (17)	0.0230 (17)	0.0000 (14)	0.0062 (15)	0.0018 (15)
C8	0.0238 (18)	0.0177 (16)	0.0227 (18)	0.0022 (14)	0.0046 (15)	0.0007 (15)
C11	0.0225 (18)	0.0206 (17)	0.032 (2)	0.0023 (14)	0.0134 (16)	0.0027 (16)
C6	0.0221 (17)	0.0197 (16)	0.0268 (18)	0.0032 (14)	0.0027 (15)	0.0045 (15)
C1	0.030 (2)	0.0181 (17)	0.0183 (17)	0.0013 (14)	0.0046 (16)	0.0034 (15)
C4	0.0172 (17)	0.0215 (17)	0.035 (2)	−0.0027 (14)	0.0082 (16)	−0.0072 (16)
C3	0.0238 (18)	0.0193 (17)	0.0235 (17)	−0.0012 (14)	0.0024 (15)	−0.0002 (15)
C7	0.0214 (18)	0.0187 (16)	0.0287 (17)	0.0021 (14)	0.0079 (16)	−0.0043 (16)
C5	0.0170 (17)	0.037 (2)	0.0290 (19)	0.0053 (15)	0.0007 (16)	−0.0096 (18)
C13	0.033 (2)	0.033 (2)	0.023 (2)	0.0073 (17)	0.0038 (16)	0.0011 (17)
C12	0.027 (2)	0.036 (2)	0.058 (3)	−0.0085 (17)	0.012 (2)	−0.012 (2)
B1	0.025 (2)	0.037 (3)	0.028 (2)	0.0001 (19)	0.0062 (18)	−0.011 (2)

### Geometric parameters (Å, º)

**Table:**

O1—H1	0.8400	C6—H6	1.0000
O1—C1	1.340 (4)	C6—C7	1.532 (5)
O2—C1	1.227 (4)	C6—C5	1.515 (4)
N1—H1A	0.9100	C1—B1	1.586 (5)
N1—H1B	0.9100	C4—C3	1.530 (4)
N1—C2	1.504 (4)	C4—C5	1.541 (4)
N1—B1	1.605 (5)	C4—C12	1.535 (4)
C9—H9A	0.9900	C3—H3A	0.9900
C9—H9B	0.9900	C3—H3B	0.9900
C9—C2	1.542 (4)	C7—H7A	0.9900
C9—C8	1.530 (4)	C7—H7B	0.9900
C2—C10	1.525 (4)	C5—H5A	0.9900
C2—C3	1.520 (4)	C5—H5B	0.9900
C10—H10A	0.9900	C13—H13A	0.9800
C10—H10B	0.9900	C13—H13B	0.9800
C10—C6	1.557 (4)	C13—H13C	0.9800
C8—C11	1.541 (4)	C12—H12A	0.9800
C8—C7	1.535 (4)	C12—H12B	0.9800
C8—C13	1.539 (5)	C12—H12C	0.9800
C11—H11A	0.9900	B1—H1C	1.19 (3)
C11—H11B	0.9900	B1—H1D	1.07 (4)
C11—C4	1.523 (5)		
			
C1—O1—H1	109.5	O2—C1—O1	118.8 (3)
H1A—N1—H1B	106.9	O2—C1—B1	125.8 (3)
C2—N1—H1A	107.3	C11—C4—C3	109.6 (3)
C2—N1—H1B	107.3	C11—C4—C5	108.6 (3)
C2—N1—B1	120.0 (3)	C11—C4—C12	109.4 (3)
B1—N1—H1A	107.3	C3—C4—C5	108.2 (3)
B1—N1—H1B	107.3	C3—C4—C12	110.1 (3)
H9A—C9—H9B	108.1	C12—C4—C5	110.8 (3)
C2—C9—H9A	109.5	C2—C3—C4	110.2 (2)
C2—C9—H9B	109.5	C2—C3—H3A	109.6
C8—C9—H9A	109.5	C2—C3—H3B	109.6
C8—C9—H9B	109.5	C4—C3—H3A	109.6
C8—C9—C2	110.7 (2)	C4—C3—H3B	109.6
N1—C2—C9	107.2 (2)	H3A—C3—H3B	108.1
N1—C2—C10	109.8 (2)	C8—C7—H7A	109.7
N1—C2—C3	110.8 (2)	C8—C7—H7B	109.7
C10—C2—C9	109.2 (2)	C6—C7—C8	109.7 (3)
C3—C2—C9	109.6 (3)	C6—C7—H7A	109.7
C3—C2—C10	110.2 (2)	C6—C7—H7B	109.7
C2—C10—H10A	110.1	H7A—C7—H7B	108.2
C2—C10—H10B	110.1	C6—C5—C4	109.9 (3)
C2—C10—C6	107.8 (3)	C6—C5—H5A	109.7
H10A—C10—H10B	108.5	C6—C5—H5B	109.7
C6—C10—H10A	110.1	C4—C5—H5A	109.7
C6—C10—H10B	110.1	C4—C5—H5B	109.7
C9—C8—C11	108.6 (3)	H5A—C5—H5B	108.2
C9—C8—C7	108.3 (3)	C8—C13—H13A	109.5
C9—C8—C13	109.6 (3)	C8—C13—H13B	109.5
C7—C8—C11	108.6 (3)	C8—C13—H13C	109.5
C7—C8—C13	111.4 (3)	H13A—C13—H13B	109.5
C13—C8—C11	110.3 (3)	H13A—C13—H13C	109.5
C8—C11—H11A	109.4	H13B—C13—H13C	109.5
C8—C11—H11B	109.4	C4—C12—H12A	109.5
H11A—C11—H11B	108.0	C4—C12—H12B	109.5
C4—C11—C8	111.3 (3)	C4—C12—H12C	109.5
C4—C11—H11A	109.4	H12A—C12—H12B	109.5
C4—C11—H11B	109.4	H12A—C12—H12C	109.5
C10—C6—H6	109.0	H12B—C12—H12C	109.5
C7—C6—C10	109.7 (3)	N1—B1—H1C	104.6 (18)
C7—C6—H6	109.0	N1—B1—H1D	107 (2)
C5—C6—C10	109.5 (3)	C1—B1—N1	106.1 (3)
C5—C6—H6	109.0	C1—B1—H1C	109.7 (16)
C5—C6—C7	110.5 (3)	C1—B1—H1D	111 (2)
O1—C1—B1	115.4 (3)	H1C—B1—H1D	118 (3)

### Hydrogen-bond geometry (Å, º)

**Table:**

*D*—H···*A*	*D*—H	H···*A*	*D*···*A*	*D*—H···*A*
O1—H1···O2^i^	0.84	1.82	2.662 (3)	176
N1—H1*B*···O1^ii^	0.91	2.11	3.011 (3)	171

Symmetry codes: (i) −*x*+1, −*y*+2, −*z*+2; (ii) *x*, −*y*+2, *z*−1/2.
