# Supplementary figures and images for: Crystal structure of memantine–carb­oxy­borane
Source: Acta Crystallogr E Crystallogr Commun. 2019 Apr 2;75(Pt 5):543–6. doi: 10.1107/S2056989019004092 (PMC6505612; doi:10.1107/S2056989019004092)

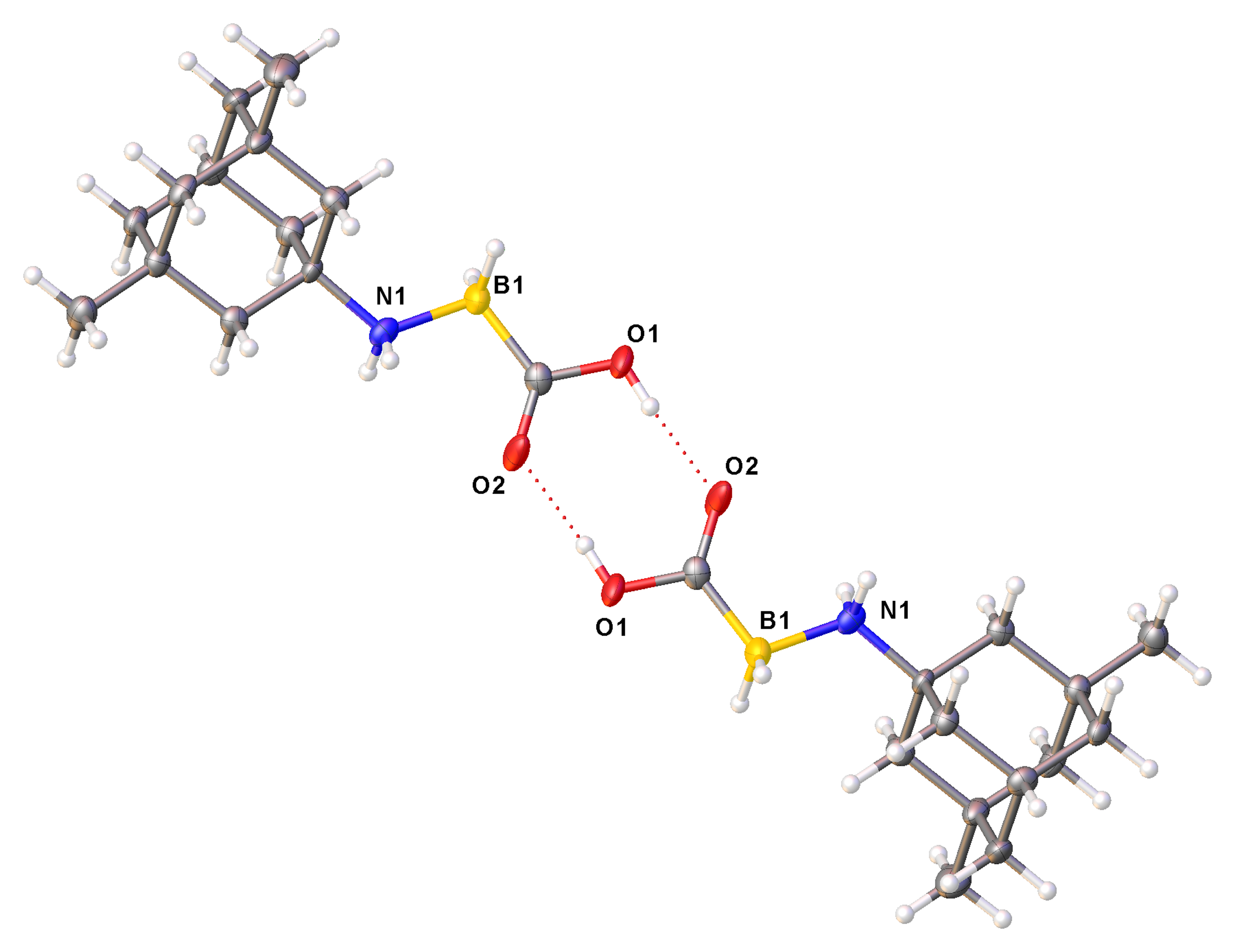

Supplement: Supplementary file 5 [file e-75-00543-sup3.tif]
